# Arbuscular mycorrhizal fungi enhance nitrogen assimilation and drought adaptability in tea plants by promoting amino acid accumulation

**DOI:** 10.3389/fpls.2024.1450999

**Published:** 2024-09-16

**Authors:** Xiao-Long Wu, Yong Hao, Wei Lu, Chun-Yan Liu, Jia-Dong He

**Affiliations:** ^1^ College of Horticulture and Gardening, Yangtze University, Jingzhou, China; ^2^ College of Urban Construction, Yangtze University, Jingzhou, China; ^3^ Earth and Life Institute, Université catholique de Louvain-UCLouvain, Louvain-la-Neuve, Belgium

**Keywords:** amino acid, arbuscular mycorrhizal fungus, drought stress, nitrogen metabolism, tea plant

## Abstract

The development and quality of tea plants (*Camellia sinensis* (L.) O. Ktze.) are greatly hampered by drought stress (DS), which affects them in a number of ways, including by interfering with their metabolism of nitrogen (N). Arbuscular mycorrhizal fungi (AMF) are known to enhance water and nutrient absorption in plants, but their specific effects on tea plant N metabolism under DS and the associated regulatory mechanisms remain unclear. This study aimed to evaluate the impact of *Claroideoglomus etunicatum* inoculation on N assimilation in tea plants (*C. sinensis* cv. Fuding Dabaicha) under well-watered (WW) and DS conditions, and to explore potential molecular mechanisms. After 8 weeks of DS treatment, root mycorrhizal colonization was significantly inhibited, and the biomass of tea shoots and roots, as well as the contents of various amino acids (AAs) were reduced. However, AMF inoculation significantly increased the contents of tea polyphenols and catechins in leaves by 13.74%-36.90% under both WW and DS conditions. Additionally, mycorrhizal colonization notably increased N content by 12.65%-35.70%, various AAs by 11.88%-325.42%, and enzymatic activities associated with N metabolism by 3.80%-147.62% in both leaves and roots. Gene expression analysis revealed a universal upregulation of N assimilation-related genes (*CsAMT1;2*, *CsAMT3;1*, *CsGS1*, *CsNADH-GOGAT*, *CsTS2*, *CsGGT1*, and *CsADC*) in AMF-colonized tea roots, regardless of water status. Under DS condition, AMF inoculation significantly upregulated the expressions of *CsNRT1;2*, *CsNRT1;5*, *CsNRT2;5*, *CsNR*, *CsGS1*, *CsGDH1*, *CsGDH2*, *CsTS2*, *CsGGT1*, *CsGGT3*, and *CsSAMDC* in tea leaves. These findings suggest that AMF improved tea plant adaptability to DS by enhancing N absorption and assimilation, accompanied by the synthesis and accumulation of various AAs, such as Glu, Gln, Asp, Lys, Arg, GABA and Pro. This is achieved through the upregulation of N metabolism-related genes and the activation of related enzymes in tea plants under DS condition. These findings provide valuable insights into the role of AMF in regulating tea plant N metabolism and enhancing stress tolerance.

## 1 Introduction

*Camellia sinensis* (L.) O. Ktze., originating from southwestern China, is highly valued economically as it is processed into one of the world’s three major non-alcoholic beverages. Consequently, tea has become an important leafy economic crop in the tropical and subtropical regions of Asia (Liu et al., 2020a). Tea plants are highly sensitive to soil moisture, and drought stress (DS) significantly inhibit their growth, affecting both quality and yield. Therefore, DS remains one of the principal challenges in tea production (Guo et al., 2017). Previous study has revealed that DS restricts plant development through inhibiting photosynthesis and respiration, reducing carbohydrate synthesis, accelerating protein hydrolysis, damaging cell membrane permeability, enhancing antioxidant metabolism, and altering the levels and distribution of hormones and amino acid (AA) (Qian et al., 2018). DS also affects the transcriptional levels of drought-responsive functional genes (Qian et al., 2018). Additionally, DS significantly inhibits the absorption and transportation of mineral elements such as nitrogen (N), phosphorus (P), potassium (K), and calcium (Ca) in tea plants (Liu et al., 2024), among which N plays a pivotal role in regulating tea plant growth and the synthesis of AAs and related secondary metabolites, which are crucial for yield and quality (Xie et al., 2023). This makes understanding N metabolism under DS critical.

Ammonium (NH_4_^+^) and nitrate (NO_3_^-^) are the primary inorganic N sources absorbed by tea roots from the soil. These compounds are transported through ammonium transporters (AMTs) and nitrate transporters (NRTs) to be utilized by plants (Zhang et al., 2023). AMTs are localized in the cell membrane, and the transcription of genes encoding these proteins is influenced by various factors, including external N levels and mycorrhizal symbiosis (Zhang et al., 2023). In tea plants, the expressions of *CsAMTs* and membrane-bound NRTs exhibit tissue-specific patterns. Specifically, *CsAMT1;2* and *CsNRT1;5* show the highest transcriptional abundance in roots, while *CsAMT1;1*, *CsAMT3;1*, *CsNRT1;2*, and *CsNRT2;5* are primarily expressed in leaves (Zhang et al., 2018, 2023). NO_3_^-^ absorbed by plants must first be reduced to nitrite (NO_2_^-^) in the cytoplasm by nitrate reductase (NR) and then further reduced to NH_4_^+^ in the plastids by nitrite reductase (NiR) (Liu et al., 2022b). In vascular plants, more than 95% of NH_4_^+^/NH_3_ is assimilated through the glutamine synthetase-glutamate synthetase (GS-GOGAT) cycle (Thomsen et al., 2014). The expression of GS- and GOGAT-related genes exhibits tissue-specific patterns in tea plants. GS is primarily involved in NH_4_^+^ assimilation and NO_3_^-^ reduction, whereas GOGAT predominantly facilitates ammonia assimilation (Zhang et al., 2023). Additionally, glutamate dehydrogenase (GDH) catalyzes the conversion of ammonia to glutamic acid (Glu) in plant tissues and deaminates Glu to α-ketoglutarate, which serves as a stress response enzyme to detoxify intracellular high ammonia (Zhang et al., 2023). Furthermore, theanine (Thea) synthesis represents a distinctive N assimilation pathway in tea plants (Lin et al., 2023). In tea roots, Thea can be synthesized by two routes: theanine synthetase (TS) from Glu and ethylamine (EA) (Zhang et al., 2023), and gamma-glutamyl transferase (GGT) from glutamine (Gln) and EA (Yang et al., 2021). In this process, EA is synthesized from alanine (Ala) through alanine decarboxylase (AlaDC) catalysis, while Ala is typically produced from pyruvate via alanine aminotransferase (ALT) (Lin et al., 2023). S-adenosylmethionine decarboxylase (SAMDC) and arginine decarboxylase (ADC) share domains with AlaDC and are often analyzed as alternative enzymes (Shi et al., 2011). Thea synthesized in roots is transported to new buds and leaves via vascular tissues for accumulation and catabolic metabolism, ultimately degrading into Glu and EA, with EA being oxidized into acetaldehyde by amine oxidase (AO), potentially contributing to catechin synthesis (Lin et al., 2023). Thus, understanding N uptake and assimilation in tea plants is essential.

Glutamic-oxaloacetic transaminase (GOT) and glutamic-pyruvic transaminase (GPT) serve as key enzymes for producing other AAs by catalyzing the conversion of Glu in plants (Hildebrandt et al., 2015). Glutamate decarboxylase (GAD) specifically catalyzes the synthesis of gamma-aminobutyric acid (GABA) from Glu (Xu et al., 2017). Asparagine synthase (ASNS) is a crucial enzyme in plant N metabolism, catalyzing the synthesis of asparagine (Asn), an essential carrier for transporting newly synthesized N within the plant and for translocating N from aging organs (Gaufichon et al., 2010). Pyruvate, a crucial intermediate of glycolysis, not only participates in energy metabolism but also closely links to amino acid synthesis through reactions such as transamination, serving as a vital bridge between carbohydrate and amino acid metabolism. Key enzymes, including phosphoenolpyruvate carboxylase (PEPC), pyruvate kinase (PK), pyruvate decarboxylase (PDC), pyruvate dehydrogenase (PDH), and pyruvate dehydrogenase kinase (PDK), play significant roles in pyruvate production, consumption, and regulation (Liu et al., 2020b). Recent studies have shown that under DS, N absorption in plants decreases, and the activity of N metabolism-related enzymes weakens, leading to lower N metabolism efficiency (Dong et al., 2022; Du et al., 2020). Therefore, enhancing drought resistance and improving tea quality by optimizing N metabolism in tea plants is particularly important.

Arbuscular mycorrhizal fungi (AMF), beneficial microorganisms in soil, infect approximately 80% of terrestrial plant roots, establishing a mutualistic symbiosis (Parniske, 2008). Research has found that AMF promote plant growth and development by rapidly supplying water and nutrients through its extensive external and intraradical hyphae (Parniske, 2008). Numerous studies have revealed that AMF enhances plant drought tolerance by accelerating nutrient acquisition, promoting leaf gas exchange, activating antioxidant defense systems, improving osmotic adjustment, and regulating the expression of drought-tolerant functional genes (Wang et al., 2023). Additionally, AMF has been shown to increase root system development, enhancing water and nutrient uptake, which contributes to better physiological performance under drought conditions ​ (Nader et al., 2024). Biochemically, AMF enhances the activities of antioxidant enzymes like catalase and peroxidase, mitigating oxidative stress and reducing lipid peroxidation​ (He et al., 2020; Sheteiwy et al., 2021). On a molecular level, AMF modulates the expression of key genes involved in proline metabolism and sugar transport, as well as aquaporin genes, which are crucial for maintaining water homeostasis, further improving the plant’s resilience to drought stress​ (Sheteiwy et al., 2021). Regarding N metabolism, previous study has reported that AMF inoculation facilitates N absorption and utilization in white clover (*Trifolium repens*), evidenced by increased N content, N metabolic enzymes activities, and AA concentrations in both leaves and roots (Xie et al., 2021). Similar results were obtained in *Catalpa bungei*. Inoculation with AMF could promote N uptake and assimilation by comprehensively regulating the expression of key enzyme genes of root nitrogen metabolism and nitrate transporter genes (Chen et al., 2023). In tea plants, the synthesis and accumulation of free AAs in AMF-colonized seedlings were notably promoted, possibly related to the upregulation of AA synthesis genes *CsGDH* and *CsGOGAT* (Shao et al., 2019; Wang et al., 2020). These processes emphasize the importance of AA synthesis under stress. These findings suggest a significant role for AMF in enhancing N metabolism.

Acknowledging the challenge posed by the conflict between tea plants’ preference for acidic soils (pH 4.5-6.0) and the optimal pH range (6.0-8.0) for N assimilation (Li et al., 2016a), which is crucial for tea growth and development, our study endeavors to fill this research gap. While AMF are known to enhance water and nutrient uptake by plants, the specific effect on N metabolism of tea plants under DS condition and the underlying regulatory mechanism remain unclear. We hypothesize that mycorrhizal colonization with *Claroideoglomus etunicatum* (*C. etunicatum*) can optimize AA accumulation and distribution in tea plants, thereby promoting their growth and development through improved N metabolism and transport under DS condition. To test this hypothesis, we inoculated tea seedlings and exposed them to both well-watered (WW) and DS conditions. The present study investigated mycorrhizal development, biomass accumulation, N and phenolic contents, amino acid composition, as well as the activity and gene expression of enzymes related to AA synthesis. Our objectives were to evaluate the effect of *C. etunicatum* inoculation on the N assimilation process of *C. sinensis* cv. Fuding Dabaicha, and to explore the potential molecular mechanism involved, providing new insights into how mycorrhizal colonization can enhance tea growth and stress resistance under drought condition.

## 2 Materials and methods

### 2.1 Experimental materials

According to Shao et al. (2018), *Claroideoglomus etunicatum* was selected as the AMF strain, provided by the Chinese Arbuscular Mycorrhizal Fungi Germplasm Bank (BGC), and propagated for 16 weeks with white clover (*Trifolium repens*). Tea seeds of *C. sinensis* cv. Fuding Dabaicha were provided by the Tea Research Institute of Guizhou Academy of Agricultural Sciences. The seeds were disinfected and germinated for 45 days following the method described by Liu et al. (2022a). Two 3-leaf-old tea seedlings of uniform size were selected and transplanted into 1.8 L plastic pots (top inner diameter: 12 cm, bottom inner diameter: 9.0 cm, height: 14 cm) filled with 1.5 kg of pre-autoclaved (0.11 MPa, 121°C, 2h) mixed substrate (soil: sand = 1:1, v/v). At the time of transplantation, 80 g of mycorrhizal inoculum (approximately 3440 spores) were added to each pot as the AMF treatment. The inoculum contained spores (43 spores/g), river sand, fungal mycelium, and root fragments. For the non-AMF treatment, an equal volume of autoclaved (0.11 MPa, 121°C, 2h) inoculum was added, along with a 2 mL filtrate (25 μm) of the inoculum to maintain a similar microbial population, except for the AM fungus. All the seedlings were placed in a greenhouse at Yangtze University (Jingzhou) with a photon flux density of 900 µmol/m^2^/s, a diurnal temperature range of 28/20°C, and a relative humidity of 80%. The pots were rearranged weekly to mitigate environmental effects.

### 2.2 Experimental design

A completely randomized design with two factors was implemented: inoculation with (+AMF) or without (-AMF) AMF, and water treatments: well-watered (WW, 75% of soil maximum water holding capacity) and drought-stressed (DS, 55% of soil maximum water holding capacity), resulting in four treatment combinations. Each treatment was replicated six times, resulting in a total of 24 pots arranged randomly.

Tea seedlings were cultivated for 4 weeks in WW condition to ensure proper mycorrhizal colonization. Then, half of the AMF and non-AMF seedlings were randomly assigned to DS condition for 8 weeks, while the others remained under WW setting. The DS-treated pots were weighed daily at 18:00, and any water lost was restored to keep the soil water content constant.

### 2.3 Biomass and mycorrhizal development

The roots and leaves of the tea plants were randomly divided into two parts at harvest. One part was oven-dried at 75°C for 48 hours after an initial treatment at 105°C and weighed, while the other part was washed and stored at −80°C. Additionally, twenty 1-cm long root segments from each seedling were cleared with 10% KOH at 95°C for 90 minutes and examined under a biological microscope after staining with 0.05% trypan blue in lactoglycerol for three minutes, as modified from Phillips and Hayman (1970). Mycorrhizal dependency, a difference in plant growth between mycorrhizal and nonmycorrhizal treatments, was calculated using plant dry matter content data following the formula described by Ma et al. (2021).

### 2.4 N content and phenolic substances content

The oven-dried root and leaf samples were ground into 0.5 mm powder. Samples were extracted using H_2_SO_4_-H_2_O_2_ solution and the total N content was determined using the SmartChem^®^ 200 Wet Chemistry Analyzer according to Guo et al. (2023). Tea polyphenols and catechins play important roles in the response of tea plants to the DS (Lv et al., 2021). The tea polyphenol content in the leaves was determined using the ferrous tartrate method, as described by de la Rosa et al. (2011), while the catechin content was quantified through the vanillin method, with catechin serving as the standard (Cao et al., 2021).

### 2.5 Amino acids content

The composition and content of various AAs in tea leaves and roots, including alanine (Ala), arginine (Arg), asparagine (Asn), aspartic acid (Asp), glutamine (Gln), glutamic acid (Glu), histidine (His), isoleucine (Ile), leucine (Leu), lysine (Lys), valine (Val), ornithine (Orn), phenylalanine (Phe), proline (Pro), threonine (Thr), tyrosine (Tyr), gamma-aminobutyric acid (GABA), serine (Ser), tryptophan (Trp), glycine (Gly), homocysteine (Hcy), and methionine (Met), were extracted as previously described by Virág et al. (2020), and determined using ultra-performance liquid chromatography (UPLC). The UPLC system consisted of an Eksigent Expert Ultra LC 100 (Eksigent, Netherlands) coupled to an AB Sciex QTrap 4500 series triple quadrupole linear ion trap mass spectrometer (Sciex, USA) in electron spray ionization (ESI) mode. Chromatographic separation was achieved using gradient elution on an Agilent Zorbax Eclipse C18 (1.7 µm, 2.1 mm × 100 mm) with a mixture of 10% formic acid methanol-H_2_O_2_ (1:1, v/v) as the eluant. Mobile phases A and B were 10% and 50% methanol water (containing 0.1% formic acid), respectively. The sample size was 5 μL, with a flow rate of 0.3 mL/min and a column oven temperature of 40°C. The ESI parameters were set as follows: turbo spray ion source at 500°C, ion spray voltage at 5500 V, collision gas at 6 psi, curtain gas at 30 psi, and atomization and auxiliary gases at 50 psi each.

Thea was extracted from the leaves and roots using 6 mol/L hydrochloric acid. One milliliter of homogenate was filled with nitrogen gas, hydrolyzed at 110°C for 18 hours, centrifuged at 12,000 rpm for five minutes, vacuum dried, dissolved in 0.5 mL of 0.1 mol/L hydrochloric acid, and centrifuged again at 12,000 rpm for five minutes. Thea content was detected using high-performance liquid chromatography (HPLC) equipped with a 2489 ultraviolet (UV)-visible detector and a reverse phase C18 column (5 µm, 250 mm × 4.6 mm, Phenomenex, Los Angeles, USA). The column oven temperature was set at 35°C and the wavelength at 254 nm. The mobile phase consisted of 0.05 mol/L sodium acetate aqueous solution (A) and a mixture of methanol, acetonitrile, and water (B, 1:3:1, v/v/v). The sample size was 10 μL with a flow rate of 1 mL/min.

### 2.6 N metabolism related enzymes activities

The activities of NR, GS, GOGAT, GDH, GOT, and GPT were determined following the method established by Dong et al. (2022). Crude enzyme extractions for GAD, PDC, PEPC, ASNS, PDH, PK, and PDK were prepared according to the instructions provided by the kit (Ke Ming Biotech Co., Ltd., Suzhou, China) and determined by enzyme-linked immunosorbent assay (ELISA) at 540, 340, 340, 540, and 505 nm. Enzymes activities were calculated based on fresh weight (FW) using the corresponding formulas.

### 2.7 Expression analysis of genes related to nitrogen assimilation

For N assimilation-related gene expression analysis, total RNA was isolated and purified from tea leaves and roots using the TaKaRa MiniBEST Universal RNA Extraction Kit (TaKaRa, Dalian, China). First-strand cDNA was synthesized using the PrimeScript™ RT reagent Kit with gDNA Eraser according to the supplier’s manual. The relative expression levels of N transporter genes (*CsAMT1;1*, *CsAMT1;2*, *CsAMT3;1*, *CsNRT1;2*, *CsNRT1;5*, *CsNRT2;5*) and AA synthesis and metabolism genes (*CsNR*, *CsNiR*, *CsGS1*, *CsGS2*, *CsNADH-GOGAT*, *CsFd-GOGAT*, *CsGDH1*, *CsGDH2*, *CsTS1*, *CsTS2*, *CsGGT1*, *CsGGT3*, *CsALT*, *CsSAMD*, *CsADC*, *CsCuAO*, *CsPAO*) were determined by qRT-PCR. Relative expression levels were measured using the CFX96 Real Time PCR Detection System (BIO-RAD, Berkeley, CA, USA) with TBP as the internal reference gene. qRT-PCR was run on a Bio-Rad CFX96 with SYBR Green I dye (Vazyme, China), with a reaction mixture of 10 μL SYBR qPCR Master Mix, 0.4 μL each of forward and reverse primers, 2 μL cDNA, and 7.2 μL ddH_2_O. Data were analyzed with Opticon monitor software (Bio-Rad). *CsTBP* was used as an internal control. All primers used for qRT-PCR are listed in **Table 1**. Each sample was analyzed in triplicate (biological replicates), and quantitative results were calculated using the 2^-ΔΔCt^ method (Livak and Schmittgen, 2001).

**Table 1:** Gene-specific primer sequences used in this study.

Gene	Sequence of Forward Primer (5′→3′)	Sequence of Reverse Primer (5′→3′)
*CsAMT1;1*	GGTTGTACGGCGCTTTCATC	GCTTCCGCTGGTGTATGGAT
*CsAMT1;2*	TATTTTCGGGTGGGTGTCGG	TTCTGAGGCTCGACCTTCCT
*CsAMT3;1*	ATCACCGGTCTCGTTTGCAT	TGTGTCGTCGATTTGCTGGA
*CsNRT1;2*	TTCATCCTCCCCATTGTGC	TGGATTGAATTGGTCGGCTC
*CsNRT1;5*	CCAGGTTCTGCCAAGTGATAGT	GCTCCCCTTTTCATTCACTACA
*CsNRT2;5*	GACAATCGACAACATTATAGCGC	AGTCTGAACCACCCACAAAGTCC
*CsNR*	TTGATGCTTGGGCTGACA	ACGGACCAGGGATGTGCT
*CsNiR*	GGACAGGCTGCCCAAATAG	TCACTCCCAATCCTCCCTC
*CsGS1*	ATCAGTTGTGGATGGCTCG	CACTTCGCATGGACTTGGTAC
*CsGS2*	GTGGCACCAACGGAGAAGT	CAAGGATGTATCTAGCGCACC
*CsNADH-GOGAT*	GCAGCGAGGAGATGATTGA	CACCTTCCACATTGGTTGAG
*CsFd-GOGAT*	TGCTGGTATGACTGGAGGTT	CAACTGCCAGAATAGCGGTA
*CsGDH1*	GAGCTGAAGACATACATGACCA	GCACGAGCAACACGATTAA
*CsGDH2*	ATGTGGGACGAAGAGAAGGTG	GCAACACGATTCACTCCCAG
*CsTS1*	AGACCGCCGACATCAACAC	ATGGCTTCCACAGCAGAGT
*CsTS2*	CCTAAACCTATTGAGGGTGACTG	TCCTGTAAGCCGACGCTCATT
*CsGGT1*	GATGAATCTTGGTGATCCTGAT	TTCCACCGTCCACCATAGT
*CsGGT3*	GGAGTCAGCTTCAAGATCACG	CAGTAGGCGTCGAGAAGTCAC
*CsALT*	CGAGTCCTACGAGTCTTATTATGC	GGAGGCGTTGACAATAGAATG
*CsSAMDC*	TTCCAGCCAAGCGAGTTC	AACCTCCCTCCTTGCCGA
*CsADC*	GGGCTTATGAGGAGGCAC	GCAAGAGGGTCCTGGCAT
*CsCuAO*	CAGGTGTTGAGGTGAATGTTA	AATCCAGTGGCGAGCAGA
*CsPAO*	GTCGGGGTGACGATACCTTAG	CACCACTTAGCGTCGACATTAT
*CsTBP*	GGCGGATCAAGTGTTGGAAGGGAG	ACGCTTGGGATTGTATTCGGCATTA

### 2.8 Statistical analysis

Data processing and graph creation were performed using Microsoft Excel 2021 (Microsoft Corporation, Redmond, WA, USA) and SigmaPlot version 10.0 software (Systat Software Inc., San Jose, CA, USA). Statistical analyses were conducted using SAS version 9.1.3 software (SAS Institute Inc., Cary, NC, USA). One-way analysis of variance (ANOVA) was employed to determine the significance of differences among treatments. Multiple comparisons were performed using Duncan’s multiple range test at a significance level of *p* < 0.05.

## 3 Results

### 3.1 Mycorrhizal development and biomass production

**Figure 1:**
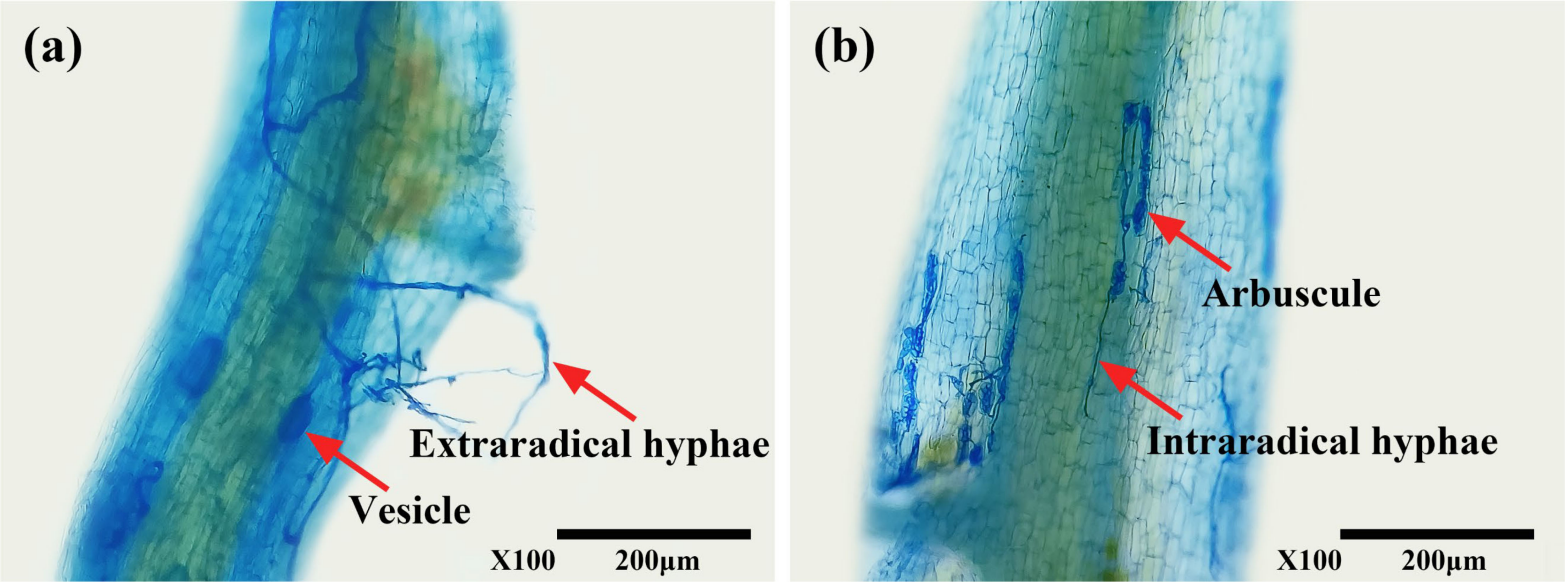
Root colonization of tea (*Camellia sinensis* cv. Fuding Dabaicha) seedlings by *C. etunicatum*. **(A)** External hyphae (external hyphae of *C. etunicatum* adhering to the root surface) and Vesicle (spherical or oval, indicating a developmental stage where nutrients and energy are stored, potentially for further colonization or reproduction); **(B)** Intraradical hyphae (intraradical hyphae of *C. etunicatum* branching within the root cortex, forming a symbiotic relationship with the host plant) and arbuscule (characterized by its highly branched structure, enhances nutrient exchange between the fungus and plant).

The presence of typical mycorrhizal structures, such as vesicles, arbuscules, and mycelium, indicated successful colonization of tea roots by AMF (**Figure 1**) under both WW and DS conditions. In contrast, non-AMF seedlings exhibited no mycorrhizal formation. Compared to WW treatment, DS treatment significantly reduced mycorrhizal colonization by 41.99% but increased mycorrhizal dependency of tea seedlings by 38.97% (**Table 2**).

**Table 2:** Effects of *C. etunicatum* on root AMF colonization and biomass of tea (*Camellia sinensis* cv. Fuding Dabaicha) leaves and roots under well-watered and drought stress.

Treatment	Mycorrhizal colonization (%)	Mycorrhizal dependence (%)	Biomass (g. DW/Plant)	
			Leaf	Root
WW-AMF	0 ± 0c	0 ± 0c	0.76 ± 0.06b	0.43 ± 0.04b
WW+AMF	29.58 ± 2.01a	22.22 ± 1.05b	0.95 ± 0.10a	0.58 ± 0.05a
DS-AMF	0 ± 0c	0 ± 0c	0.45 ± 0.05d	0.24 ± 0.01d
DS+AMF	17.16 ± 1.30b	30.88 ± 2.64a	0.64 ± 0.07c	0.36 ± 0.03c

Data (means ± SE, n = 6) followed by different lowercase letters among treatments indicate significant differences at p < 0.05.

WW, well-watered; DS, drought stress; -AMF, non-AMF inoculation; +AMF, AMF inoculation; DW, dry weight.

**Figure 2:**
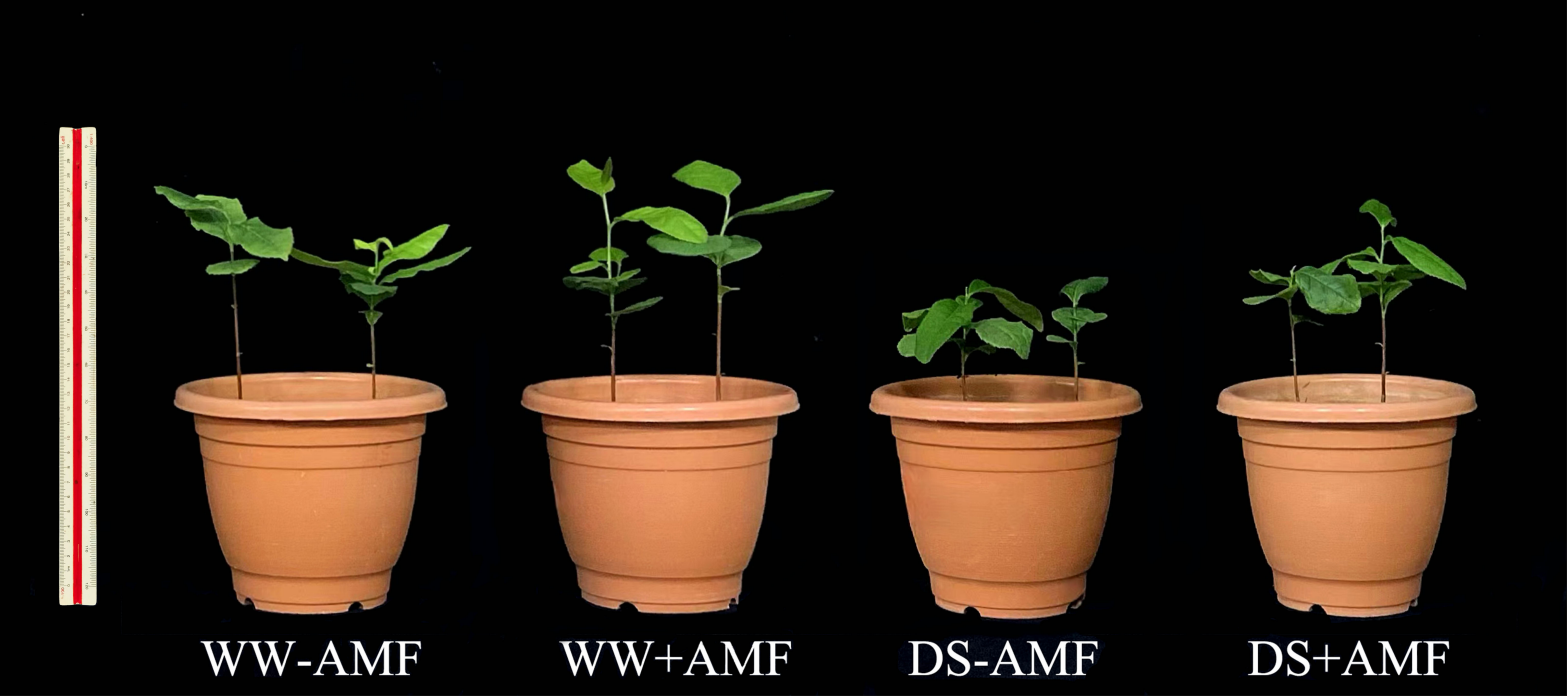
Effects of *C. etunicatum* inoculation on the growth of tea (*Camellia sinensis* cv. Fuding Dabaicha) under well-watered and drought stress. WW, well-watered; DS, drought stress; -AMF, non-AMF inoculation; +AMF, AMF inoculation.

Under DS treatment, the dry biomass of both leaves and roots were significantly decreased in tea seedlings compared to WW treatment (**Figure 2**). However, AMF treatment significantly increased biomass production in leaves and roots by 25.00% and 34.88% under WW condition, and 42.22% and 50.00% under DS condition, respectively (**Table 2**).

These findings indicated that AMF colonization effectively enhanced biomass production in tea seedlings, particularly under DS conditions.

### 3.2 Leaf phenolic substances

**Figure 3:**
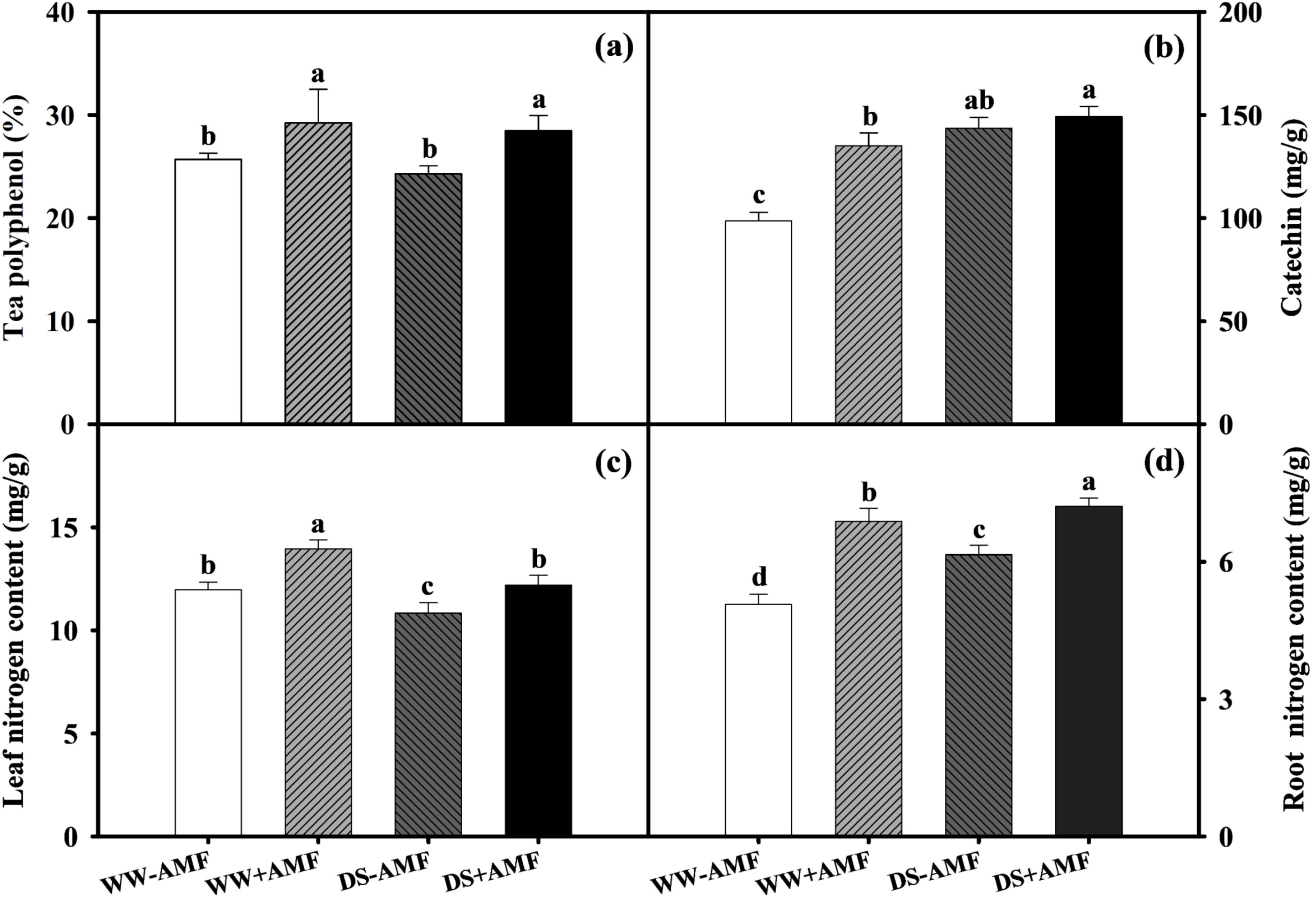
Effects of *C. etunicatum* inoculation on the phenolic compounds and nitrogen content of tea (*Camellia sinensis* cv. Fuding Dabaicha) leaves and roots under well-watered and drought stress. **(A)** Leaf tea polyphenol content. **(B)** Leaf catechin content. **(C)** Leaf nitrogen content. **(D)** Root nitrogen content. Different lowercase letters indicate significant difference within the same column at 0.05 level by LSD.

Relative to WW, DS did not affect tea polyphenol concentration (**Figure 3A**) but significantly increased catechin content by 45.45% and 10.52% (**Figure 3B**) in both AMF and non-AMF treatments. Compared to the non-AMF treatment, AMF inoculation significantly increased tea polyphenol content by 13.74% and 17.20% under WW and DS conditions, respectively, and increased catechin content by 36.90% under WW condition, with no significant effect under DS condition. This suggested that AMF inoculation selectively enhanced specific phenolic compounds in tea leaves, contributing to potential variations in tea quality under different water conditions.

### 3.3 Leaf and root nitrogen content

Total N concentrations in tea seedlings were influenced by AMF inoculation and soil water status. DS treatment generally decreased leaf N content by 9.52% and 12.54% (**Figure 3C**) but significantly increased root N content by 21.30% and 4.80% in non-AMF and AMF seedlings, respectively (**Figure 3D**). Additionally, AM symbiosis notably increased N content by 16.54% and 12.65% in leaves and by 35.70% and 17.24% in roots under WW and DS conditions, respectively. Overall, AMF inoculation improved N uptake and distribution in tea seedlings, especially under DS, which enhanced the plant’s adaptive capacity.

### 3.4 Leaf and root amino acids content

**Figure 4:**
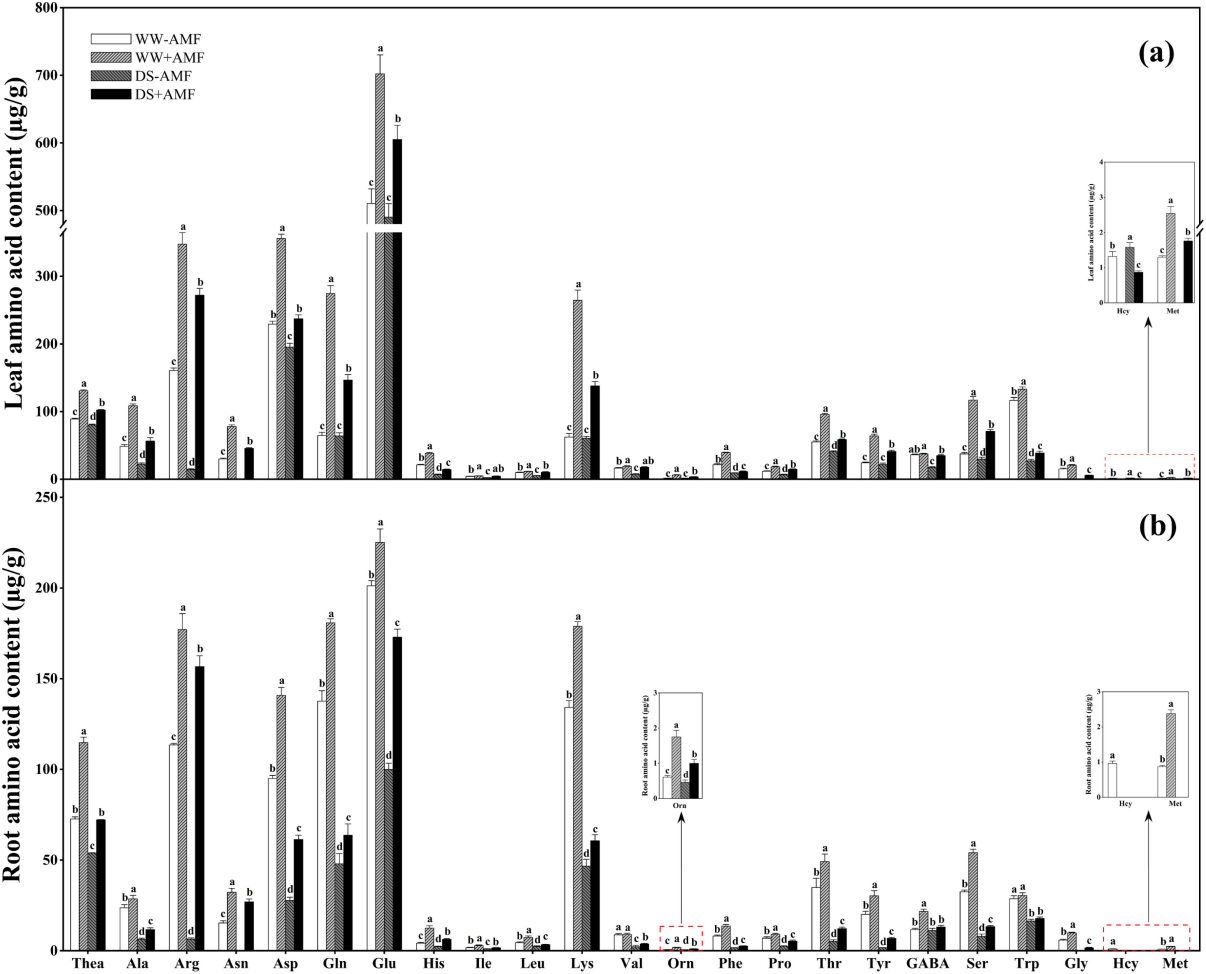
Effect of *C. etunicatum* inoculation on amino acid content (µg/g) of tea (*Camellia sinensis* cv. Fuding Dabaicha) leaves and roots under well-watered and drought stress. **(A)** Leaf amino acid content. **(B)** Root amino acid content. Different lowercase letters indicate significant difference within the same column at 0.05 level by LSD. Blank spaces indicate not detected. Thea, theanine; Ala, alanine; Arg, arginine; Asn, asparagine; Asp, aspartic acid; Gln, glutamine; Glu, glutamic acid; His, histidine; Ile, isoleucine; Leu, leucine; Lys, lysine; Val, valine; Orn, ornithine; Phe, phenylalanine; Pro, proline; Thr, threonine; Tyr, tyrosine; GABA, gamma-aminobutyric acid; Ser, serine; Trp, tryptophan; Gly, glycine; Hcy, homocysteine; Met, methionine.

DS treatment reduced the contents of Thea, Ala, Arg, Asn, Asp, His, Leu, Phe, Pro, Thr, GABA, Ser, Trp, Gly, and Met while increasing the content of Hcy in leaves, regardless of mycorrhizal colonization (**Figure 4A**). DS treatment significantly decreased Ile and Val contents under non-AMF treatment and Gln, Glu, Lys, Orn, and Tyr contents under AMF treatment. However, AMF colonization increased the contents of Thea, Ala, Arg, Asn, Asp, Gln, Glu, His, Ile, Leu, Lys, Val, Orn, Phe, Pro, Thr, Tyr, Ser, Trp, Gly, and Met but decreased Hcy content in leaves regardless of water conditions. Additionally, under DS condition, GABA accumulation was significantly enhanced by AMF inoculation.

In roots, DS treatment generally reduced the concentrations of Thea, Ala, Arg, Asn, Asp, Gln, Glu, His, Ile, Leu, Lys, Val, Orn, Phe, Pro, Thr, Tyr, Ser, Trp, Gly, and Met regardless of mycorrhizal colonization (**Figure 4B**). DS treatment also significantly reduced GABA content in AMF-colonized roots and Hcy content in non-AMF-colonized roots. Compared to non-AMF treatment, AMF colonization significantly increased root contents of Thea, Ala, Arg, Asn, Asp, Gln, Glu, His, Ile, Leu, Lys, Orn, Phe, Pro, Thr, Tyr, Ser, and Gly under WW and DS conditions. Additionally, AMF-colonized seedlings exhibited significantly higher GABA and Met concentrations under WW condition and Val concentrations under DS condition. AMF symbiosis slightly reduced Hcy content under WW condition.

These results suggested that AMF symbiosis mitigated the negative effects of DS on AA profiles in tea seedlings, potentially aiding in stress tolerance.

### 3.5 Leaf and root N metabolism-related enzymes activities

**Figure 5:**
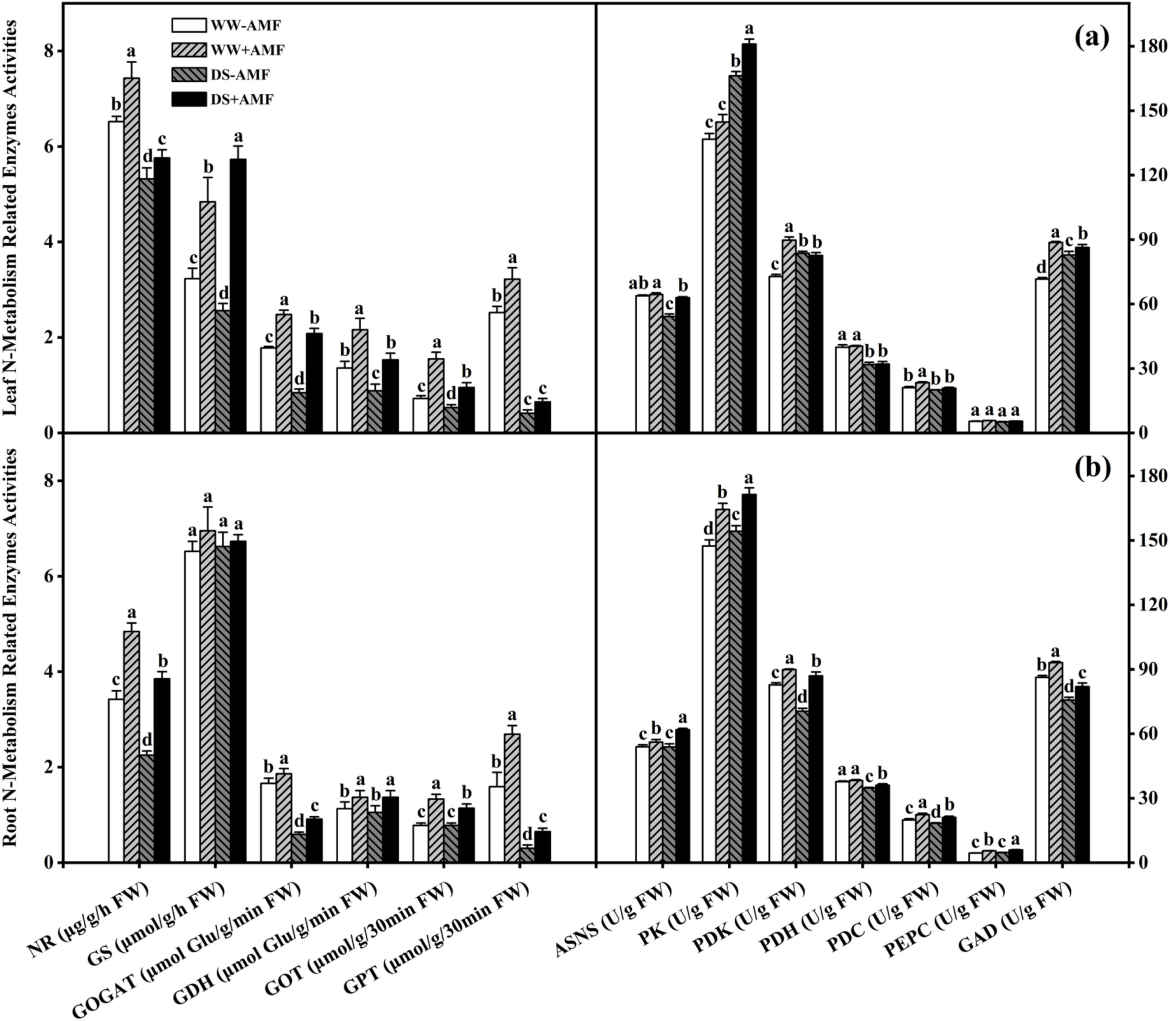
Effect of *C. etunicatum* inoculation on various N metabolism-related enzymes activities of tea (*Camellia sinensis* cv. Fuding Dabaicha) plants under well-watered and drought stress. **(A)** Leaf N metabolism-related enzymes activities. **(B)** Leaf N metabolism-related enzymes activities. Different lowercase letters indicate significant difference within the same column at 0.05 level by LSD. NR, nitrate reductase; GS, glutamine synthetase; GOGAT, glutamate Synthase; GDH, glutamate dehydrogenase; GOT, glutamic oxaloacetic transaminase; GPT, glutamic pyruvic transaminase; ASNS, asparagine synthetase; PK, pyruvate kinase; PDK, pyruvate dehydrogenase kinase; PDH, pyruvate dehydrogenase; PEPC, phosphoenolpyruvate carboxylase; GAD, glutamate decarboxylase; FW, fresh weight. The unit of measurement for each enzyme activity varies according to the method.

In leaves, DS treatment significantly reduced the activities of NR, GS, GOGAT, GDH, GOT, GPT, ASNS, and PDH in non-AMF seedlings; and it also reduced the activities of NR, GOGAT, GDH, GOT, GPT, ASNS, PDK, PDH, PDC, and GAD in AMF-colonized seedlings. However, DS treatment significantly increased the activities of PK, PDK, and GAD in non-AMF seedlings, and the activities of GS and PK in AMF-colonized seedlings. Compared to non-AMF treatment, AMF-treated seedlings recorded significantly higher activities of NR (13.96%), GS (49.85%), GOGAT (39.33%), GDH (58.82%), GOT (115.28%), GPT (27.78%), PDK (23.24%), PDC (10.92%), and GAD (23.76%) under WW condition, and considerably higher activities of NR (8.27%), GS (123.83%), GOGAT (147.62%), GDH (73.86%), GOT (79.25%), ASNS (16.14%), PK (8.85%), and GAD (4.30%) under DS condition, respectively (**Figure 5A**).

In roots, DS treatment significantly reduced the activities of NR, GOGAT, GPT, PDK, PDH, PDC, and GAD compared to WW, regardless of mycorrhizal colonization status. Additionally, DS treatment decreased GOT activity in AMF-colonized seedlings, whereas it significantly increased PK activity in non-AMF seedlings and ASNS, PK, and PEPC activities in AMF-colonized seedlings. Nevertheless, AMF symbiosis significantly enhanced the activities of NR, GOGAT, GDH, GOT, GPT, ASNS, PK, PDK, PDC, PEPC, and GAD. Specifically, the activities increased by 41.52%, 12.05%, 21.24%, 70.51%, 69.18%, 4.08%, 11.53%, 8.57%, 12.42%, 22.05%, and 7.92% under WW condition, and by 71.11%, 54.24%, 30.48%, 46.15%, 116.67%, 14.95%, 11.11%, 23.42%, 15.04%, 26.82%, and 8.30% under DS condition, respectively. Meanwhile, AMF symbiosis also significantly enhanced PDH activity by 3.80% under DS condition but did not significantly affect GS activity, irrespective of water conditions (**Figure 5B**).

Thus, AMF colonization significantly modulated N metabolism-related enzyme activities, which may have contributed to enhanced N utilization and stress resilience in tea plants.

### 3.6 Relative expression of N transporter genes

**Figure 6:**
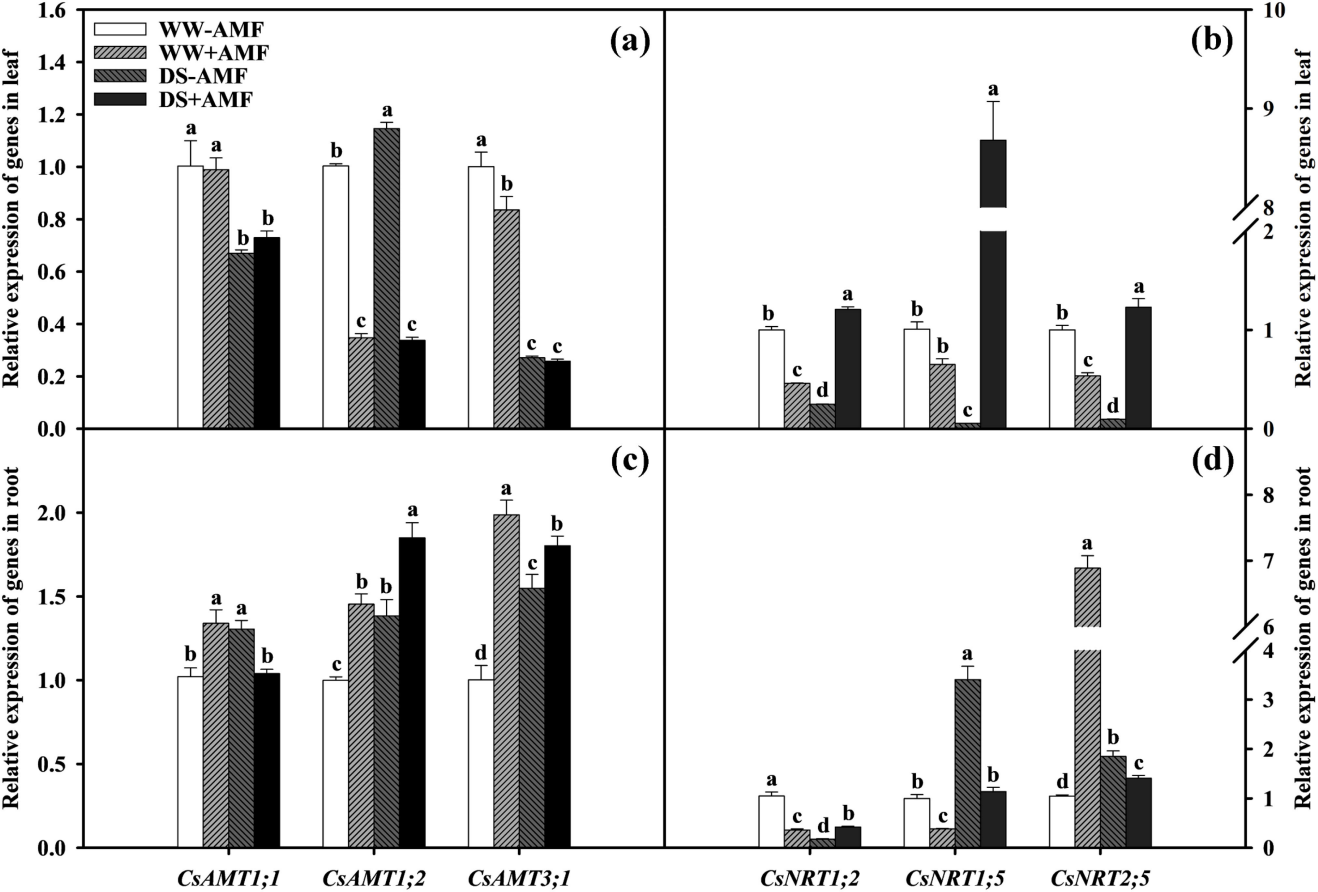
Effect of inoculation with *C. etunicatum* under well-watered and drought stress on the relative expression of *CsAMTs* **(A)** and *CsNRTs* **(B)** in leaves and *CsAMTs* **(C)** and *CsNRTs* **(D)** in roots of tea (*Camellia sinensis* cv. Fuding Dabaicha) plants. Different lowercase letters indicate significant difference within the same column at 0.05 level by LSD.

Compared with WW treatment, DS treatment significantly downregulated the expressions of *CsAMT1;1*, *CsAMT3;1*, *CsNRT1;2*, *CsNRT1;5*, and *CsNRT2;5* in leaves of non-AMF seedlings and *CsAMT1;1* and *CsAMT3;1* in leaves of AMF-colonized seedlings (**Figures 6A, B**). DS treatment significantly upregulated the expressions of leaf *CsAMT1;2* in non-AMF seedlings and *CsNRT1;2*, *CsNRT1;5*, and *CsNRT2;5* in AMF-colonized seedlings by 1.14-fold, 2.62-fold, 13.33-fold, and 2.30-fold, respectively. Compared to non-AMF treatment, AMF treatment significantly upregulated the expressions of leaf *CsNRT1;2*, *CsNRT1;5*, and *CsNRT2;5* by 4.90-fold, 161.00-fold, and 12.78-fold under DS condition, respectively. However, the expressions of leaf *CsAMT1;2*, *CsAMT3;1*, *CsNRT1;2*, and *CsNRT2;5* under WW condition and *CsAMT1;2* under DS condition was dramatically downregulated (**Figures 6A, B**).

In roots, DS treatment notably upregulated the expressions of *CsAMT1;1*, *CsAMT1;2*, *CsAMT3;1*, *CsNRT1;5*, and *CsNRT2;5* by 1.28-fold, 1.38-fold, 1.55-fold, 3.40-fold, and 1.77-fold under non-AMF treatment, and *CsAMT1;2*, *CsNRT1;2*, and *CsNRT1;5* by 1.27-fold, 1.17-fold, and 2.96-fold under AMF treatment, respectively. However, the expressions of *CsNRT1;2* in non-AMF treatment and *CsAMT1;1*, *CsAMT3;1*, and *CsNRT2;5* in AMF treatment seedlings were evidently decreased under DS treatment compared to WW treatment (**Figures 6C, D**). Compared to non-AMF treatment, AMF inoculation significantly upregulated the expressions of *CsAMT1;1*, *CsAMT1;2*, *CsAMT3;1*, and *CsNRT2;5* by 1.31-fold, 1.45-fold, 1.98-fold, and 6.58-fold, while significantly downregulating the expressions of *CsNRT1;2* and *CsNRT1;5* by 2.89-fold and 2.60-fold under WW condition. Under DS condition, AMF inoculation significantly upregulated the expressions of *CsAMT1;2*, *CsAMT3;1*, and *CsNRT1;2* by 1.34-fold, 1.16-fold, and 2.38-fold, but downregulated the expressions of *CsAMT1;1*, *CsNRT1;5*, and *CsNRT2;5* by 1.26-fold, 2.99-fold, and 1.31-fold, respectively.

These changes in gene expression highlighted the role of AMF in regulating N transport under varying water conditions, potentially optimizing nutrient uptake efficiency.

### 3.7 Relative expression of N metabolism related enzyme genes

**Figure 7:**
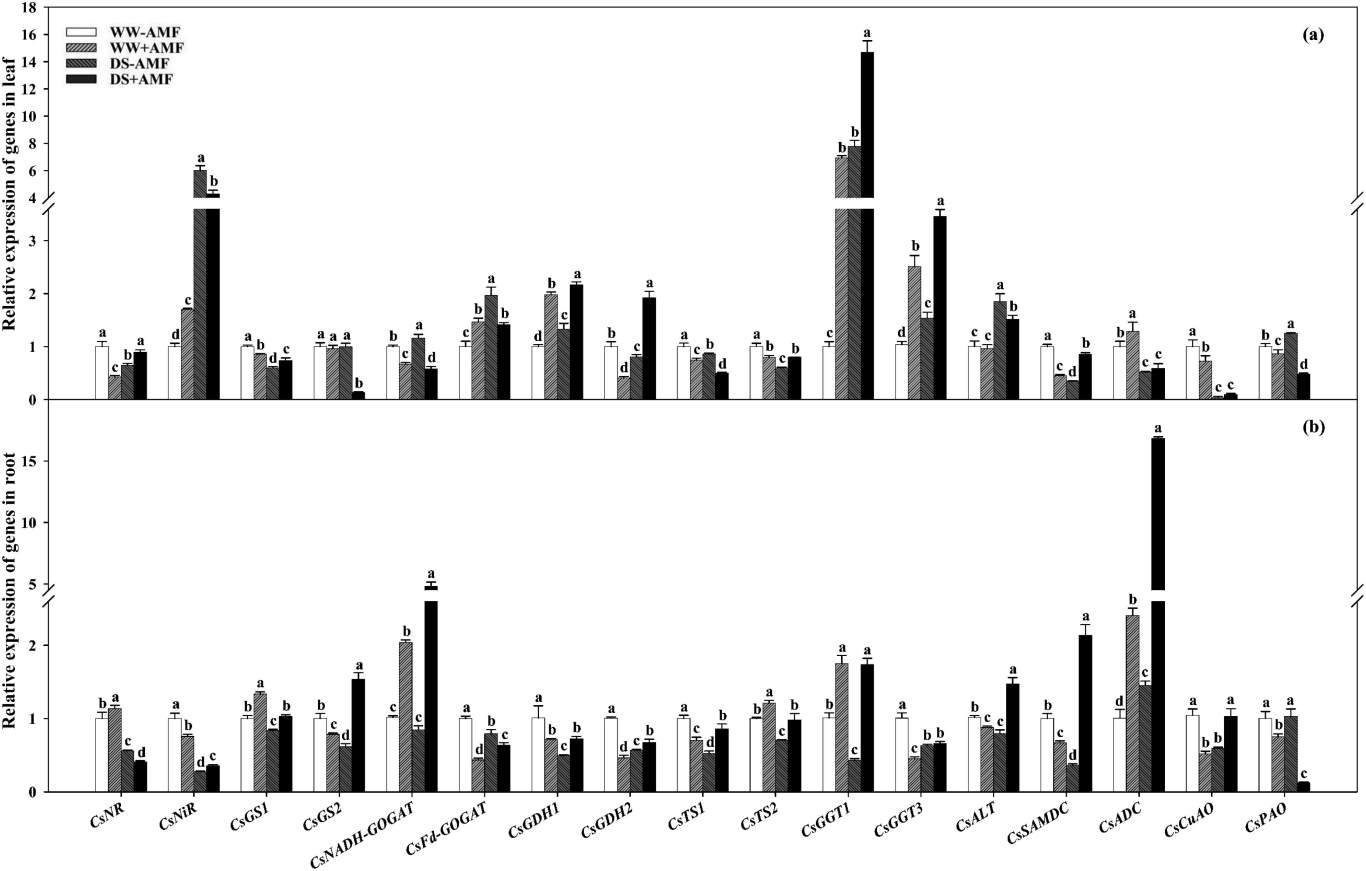
Effect of *C. etunicatum* inoculation on gene expression of enzymes related to nitrogen metabolism in leaves **(A)** and roots **(B)** of tea (*C. sinensis* cv. Fuding Dabaicha) under well-watered and drought stress. Different lowercase letters indicate significant difference within the same column at 0.05 level by LSD.

The expressions of N metabolism-related enzyme genes in leaves and roots significantly changed after AMF inoculation, regardless of water status (**Figures 7A, B**).

In leaves, DS treatment significantly downregulated the expressions of *CsNR*, *CsGS1*, *CsGDH2*, *CsTS1*, *CsTS2*, *CsSAMDC*, *CsADC*, and *CsCuAO* by 1.54-, 1.68-, 1.24-, 1.16-, 1.67-, 2.86-, 1.95-, and 25.62-fold in non-AMF seedlings and *CsGS1*, *CsGS2*, *CsNADH-GOGAT*, *CsTS1*, *CsADC*, *CsCuAO*, and *CsPAO* by 1.16-, 6.96-, 1.16-, 1.49-, 2.18-, 7.39-, and 1.80-fold in AMF seedlings, respectively. Simultaneously, DS treatment dramatically upregulated the expressions of *CsNiR*, *CsNADH-GOGAT*, *CsFd-GOGAT*, *CsGDH1*, *CsGGT1*, *CsGGT3*, *CsALT*, and *CsPAO* by 6.00-, 1.16-, 1.97-, 1.33-, 7.76-, 1.48-, 1.85-, and 1.25-fold in non-AMF treatment and *CsNR*, *CsNiR*, *CsGDH1*, *CsGDH2*, *CsGGT1*, *CsGGT3*, *CsALT*, and *CsSAMDC* by 2.09-, 2.53-, 1.09-, 4.69-, 2.12-, 1.38-, 1.58-, and 1.91-fold in AMF treatment, respectively (**Figure 7A**).

Compared with the uninoculated treatment, AMF inoculation increased the expressions of *CsNiR*, *CsFd-GOGAT*, *CsGDH1*, *CsGGT1*, *CsGGT3*, and *CsADC* by 1.70-, 1.46-, 1.98-, 6.91-, 2.42-, and 1.28-fold, respectively, and decreased the expressions of *CsNR*, *CsGS1*, *CsNADH*-*GOGAT*, *CsGDH2*, *CsTS1*, *CsTS2*, *CsSAMDC*, *CsCuAO* and *CsPAO* by 2.34-, 1.17-, 1.49-, 2.44-, 1.34-, 1.26-, 2.22-, 1.38-, and 1.16-fold under WW condition. Simultaneously, AMF colonization significantly upregulated the expressions of *CsNR*, *CsGS1*, *CsGDH1*, *CsGDH2*, *CsTS2*, *CsGGT1*, *CsGGT3*, and *CsSAMDC* by 1.38-, 1.24-, 1.63-, 2.39-, 1.33-, 1.89-, 2.24-, and 2.45-fold while downregulating the expressions of *CsNiR*, *CsGS2*, *CsNADH-GOGAT*, *CsFd-GOGAT*, *CsTS1*, *CsALT*, and *CsPAO* by 1.39-, 7.23-, 2.00-, 1.39-, 1.72-, 1.22-, and 2.61-fold under DS condition, respectively (**Figure 7A**).

In the roots of plants not inoculated with AMF, DS treatment versus WW treatment significantly decreased the expressions of *CsNR*, *CsNiR*, *CsGS1*, *CsGS2*, *CsFd-GOGAT*, *CsGDH1*, *CsGDH2*, *CsTS1*, *CsTS2*, *CsGGT1*, *CsGGT3*, *CsALT*, *CsSAMDC*, and *CsCuAO* by 1.79-, 3.66-, 1.19-, 1.63-, 1.26-, 2.04-, 1.77-, 1.90-, 1.42-, 2.35-, 1.59-, 1.28-, 2.78-, and 1.77-fold, respectively, whereas the expression of *CsADC* increased by 1.45-fold. In roots of AMF-inoculated plants, DS treatment versus WW treatment upregulated the expressions of *CsGS2*, *CsNADH-GOGAT*, *CsFd-GOGAT*, *CsGDH2*, *CsTS1*, *CsGGT3*, *CsALT*, *CsSAMDC*, *CsADC*, and *CsCuAO* by 1.95-, 2.37-, 1.45-, 1.45-, 1.22-, 1.46-, 1.68-, 3.19-, 7.00-, and 1.98-fold, respectively, while downregulating the expressions of *CsNR*, *CsNiR*, *CsGS1*, *CsTS2*, and *CsPAO* by 2.79-, 2.12-, 1.30-, 1.24-, and 5.94-fold, respectively (**Figure 7B**).

Additionally, AMF inoculation upregulated the expression levels of *CsNR*, *CsGS1*, *CsNADH-GOGAT*, *CsTS2*, *CsGGT1*, and *CsADC* under WW condition by 1.13-, 1.33-, 2.01-, 1.21-, 1.74-, and 2.40-fold, respectively, whereas the expression levels of *CsNiR*, *CsGS2*, *CsFd-GOGAT*, *CsGDH1*, *CsGDH2*, *CsTS1*, *CsGGT3*, *CsALT*, *CsSAMDC*, *CsCuAO*, and *CsPAO* were downregulated by 1.32-, 1.28-, 2.29-, 1.42-, 2.17-, 1.43-, 2.23-, 1.16-, 1.50-, 2.01-, and 1.34-fold, respectively. Under DS condition, AMF inoculation upregulated the expressions of *CsNiR*, *CsGS1*, *CsGS2*, *CsNADH-GOGAT*, *CsGDH1*, *CsGDH2*, *CsTS1*, *CsTS2*, *CsGGT1*, *CsALT*, *CsSAMDC*, *CsADC*, and *CsCuAO* by 1.31-, 1.23-, 2.49-, 5.69-, 1.46-, 1.19-, 1.63-, 1.39-, 4.05-, 1.86-, 5.94-, 11.59-, and 1.74-fold, respectively, while downregulating the expressions of *CsNR*, *CsFd-GOGAT*, and *CsPAO* by 1.38-, 1.25-, and 8.15-fold, respectively (**Figure 7B**).

The differential expression of these genes underscored the impact of AMF on N metabolism pathways, enhancing the adaptability of tea plants to DS.

### 3.8 Principal component analysis

**Figure 8:**
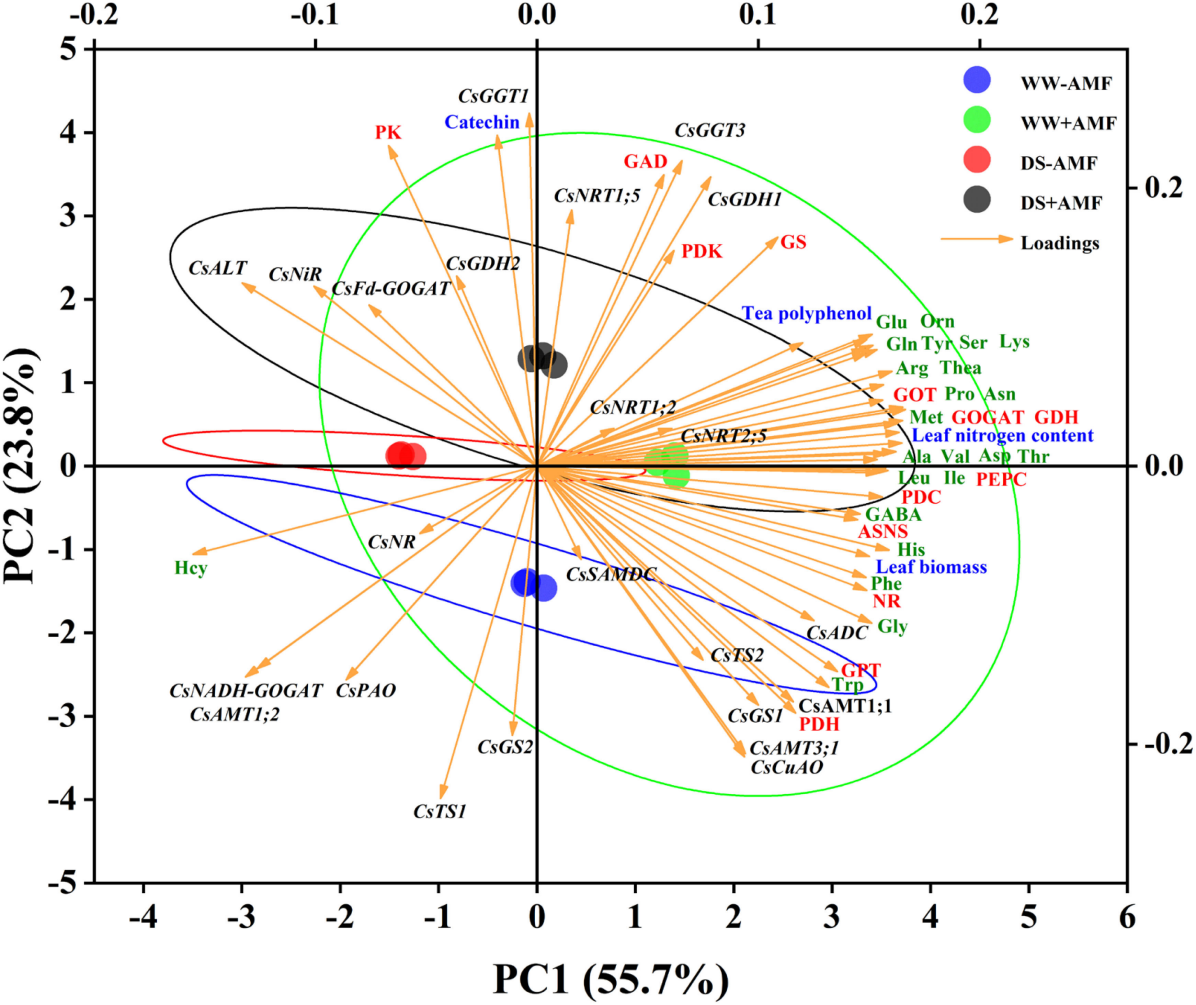
Unsupervised principal component analysis of physiological and molecular parameters of selected leaves of tea (*C. sinensis* cv. Fuding Dabaicha) inoculated with *C. etunicatum* under well-watered and drought stress.

Utilizing Principal Component Analysis (PCA), this study examined the correlations between N metabolism-related genes and major factors (biomass, N content, phenolic content, AAs levels, and enzymes activities) in both tea leaves and roots. For leaves (**Figure 8**), the cumulative contribution of the two principal components (PC1 and PC2) was 79.5% (55.7% and 23.7%, respectively), displaying good intra-group consistency and substantial inter-group variations. The expressions of certain genes (*CsAMT1;1*, *CsAMT3;1*, *CsTS2*, *CsGS1*, *CsADC*, *CsCuAO*), leaf biomass, N content, tea polyphenol content, most AAs (excluding Hcy), and enzymes activities (NR, GOGAT, GDH, GOT, GPT, ASNS, PEPC, PDC, PDH) had significant positive impacts on PC1. Notably, the expressions of some genes (*CsFd-GOGAT*, *CsNiR*, *CsALT*, *CsNR*, *CsNADH-GOGAT*, *CsAMT1;2*) showed negative correlations with these variables. Additionally, catechin content, the activities of specific enzymes (GS, GAD, PK, PDK), and the expressions of genes (*CsNRT1;5*, *CsGDH1*, *CsGDH2*, *CsGGT1*, *CsGGT3*) had substantial positive effects on PC2, while the expressions of *CsGS2* and *CsTS1* exhibited negative correlations.

**Figure 9:**
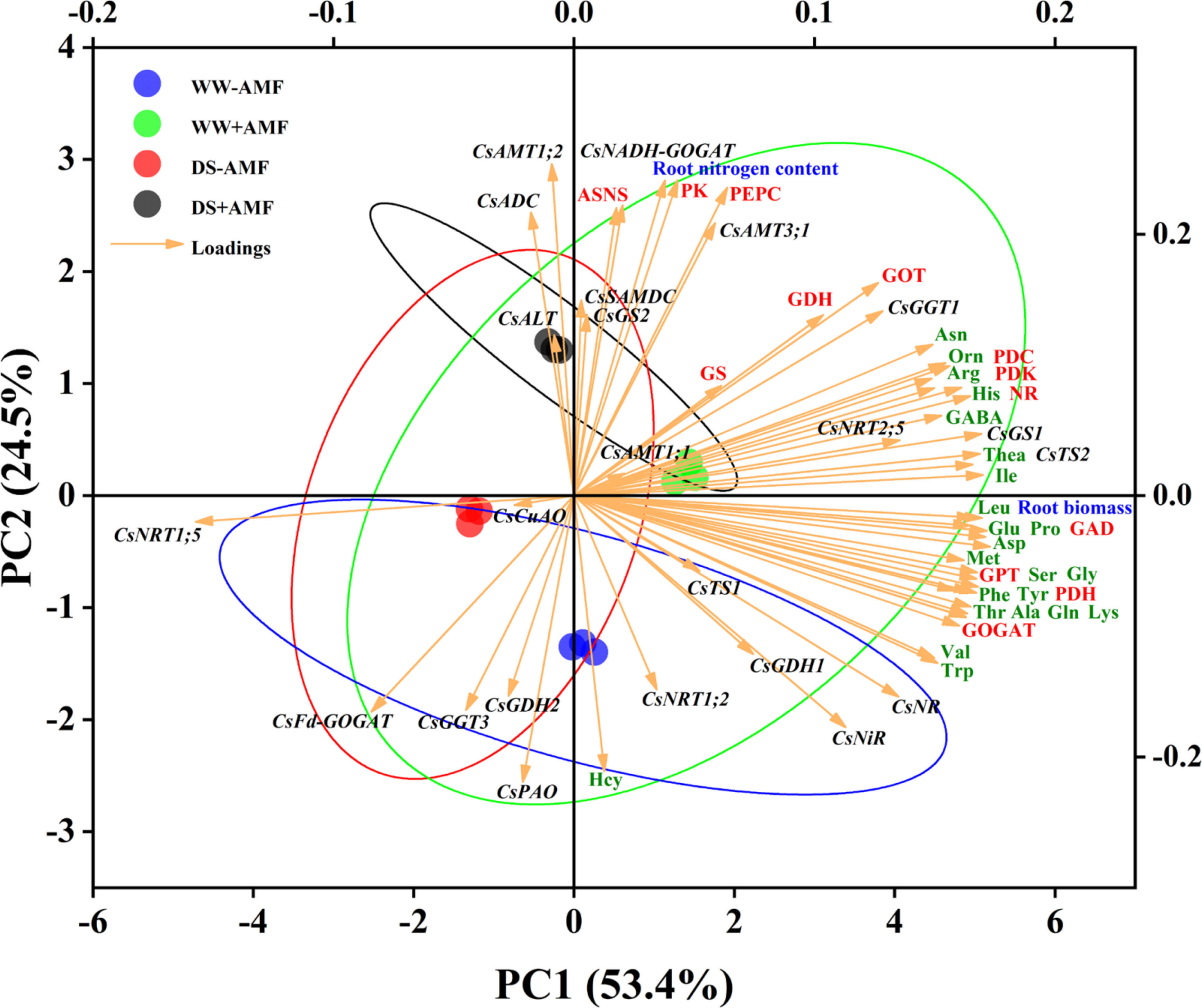
Unsupervised principal component analysis of physiological and molecular parameters of selected roots of tea (*C. sinensis* cv. Fuding Dabaicha) inoculated with *C. etunicatum* under well-watered and drought stress.

In roots (**Figure 9**), the cumulative contribution of the two principal components was 77.9% (53.4% for PC1 and 24.5% for PC2), also showing good intra-group reproducibility and inter-group differences. The expressions of genes (*CsTS2*, *CsGS1*, *CsGGT1*, *CsNRT2;5*, *CsNR*, *CsNiR*), root biomass, most AAs (excluding Hcy), and enzymes activities (NR, GOGAT, GDH, GOT, GPT, PDC, PDH, PDK, GAD) positively influenced PC1. Notably, the expressions of CsFd-GOGAT and CsNRT1;5 negatively correlated with these variables. Furthermore, root nitrogen content, activities of enzymes (ASNS, PEPC, PK), and the expressions of genes (*CsAMT1;2*, *CsAMT3;1*, *CsNADH-GOGAT*, *CsADC*) had pronounced positive effects on PC2, whereas the expressions of *CsNRT1;2*, *CsFd-GOGAT*, *CsGDH2*, *CsGGT3*, and *CsPAO* showed negative correlations.

Overall, the PCA results emphasized the complex interplay between gene expression, enzyme activities, and N metabolism, further illustrating the beneficial effects of AMF on tea plant physiology under stress conditions.

## 4 Discussion

### 4.1 Mycorrhizal development and plant growth physiology

The present study demonstrated that DS treatment significantly inhibited mycorrhizal colonization in tea seedlings (**Figure 1**; **Table 2**), consistent with previous findings (Liu et al., 2020a). This suggests that soil water scarcity may suppress spore germination and extraradical hyphae extension, thereby limiting mycorrhizal development (Huang et al., 2017). However, the mycorrhizal dependency of tea seedlings increased significantly under DS condition (**Table 2**), similar to results reported in barley by Li et al. (2014). This could be due to the hindered nutrient absorption capacity of tea roots under DS condition, making them more reliant on AMF for the acquisition of essential minerals like P, K, Ca, and Mg (Liu et al., 2024). Additionally, AMF inoculation effectively mitigated the inhibitory effects of DS treatment on biomass accumulation in tea leaves and roots (**Figure 2**; **Table 2**), aligning with the findings of Liu et al. (2024). These results reinforce the role of AMF in promoting plant growth and enhancing drought resistance (Wang et al., 2023).

In the present study, DS treatment markedly increased catechin accumulation in tea leaves (**Figure 3B**), regardless of mycorrhizal colonization, suggesting that plants may enhance drought resistance by increasing catechin content to eliminate excessive reactive oxygen species (ROS) under DS condition (Lv et al., 2021). Furthermore, AMF symbiosis notably increased tea polyphenol and catechin levels in leaves under both water conditions (**Figures 3A, B**), consistent with findings by Wang et al. (2020). This suggests that AMF may mitigate ROS-induced damage by promoting phenolic compound accumulation, thereby enhancing drought resistance and tea quality.

### 4.2 Changes in N absorption and transportation

N is a critical nutrient and limiting factor in plant growth and development. In tea plants, N content directly affects quality. Our study showed that DS treatment significantly reduced N content in tea leaves while increasing it in roots, regardless of mycorrhizal inoculation (**Figures 3C, D**). This may be due to a higher N absorption rate by the roots under DS treatment compared to the transfer rate from roots to leaves, resulting in more N concentration in the roots (He et al., 2022). The distribution of N in leaves and roots might be related to the expression of N transporter genes. The results of PCA showed that *CsAMT1;1*, *CsAMT3;1*, *CsNRT1;2*, *CsNRT2;5* were positively correlated with leaf N content (**Figure 8**), and *CsAMT1;2*, *CsAMT3;1* were positively correlated with root N content (**Figure 9**). DS treatment markedly downregulated the expressions of leaf *CsAMT1;1* and *CsAMT3;1* in both mycorrhizal and non-mycorrhizal seedlings, and leaf *CsNRT1;2*, *CsNRT1;5*, and *CsNRT2;5* in AMF-colonized seedlings (**Figures 6A, B**), potentially suppressing photorespiratory ammonia metabolism in leaves (Zhang et al., 2018) and impeding NO_3_^-^ transport from roots to xylem (Lin et al., 2008), ultimately leading to reduced leaf N content. Meanwhile, DS treatment upregulated the expressions of root *CsAMT1;2* (**Figure 6C**), enhancing the absorption of NH_4_^+^ from the soil (Zhang et al., 2023). Notably, AMF inoculation increased N content in both leaves and roots of tea seedlings regardless of water status, consistent with previous studies (Shao et al., 2018; Liu et al., 2024). This increase was accompanied by the upregulated expressions of *CsAMT1;2* and *CsAMT3;1* in roots (**Figure 6C**), indicating enhanced root absorption and transportation of NH_4_^+^ due to these genes’ upregulation (Zhang et al., 2023). However, mycorrhizal colonization significantly downregulated *CsNRT1;5* expression in tea roots (**Figure 6D**), similar to observations in *Catalpa bungei* seedlings, suggesting that mycorrhizal symbiosis may inhibit the direct absorption of NO_3_^-^ by plant roots (Chen et al., 2023).

### 4.3 Mycorrhizal colonization promote the accumulation of AAs

AAs are crucial for tea quality, balancing the astringency and bitterness of catechins and caffeine (Li et al., 2019). Previous studies have shown varying effects of DS treatment on AA contents in tea plants (Wang et al., 2016). This study found that DS treatment decreased the contents of 22 kinds of AAs (except Hcy) in both leaves and roots (**Figure 4**), which may be associated with differing drought tolerance among tea varieties (Qian et al., 2018). AMF colonization increased AAs contents in both leaves and roots under both water conditions (**Figure 4**), consistent with findings in white clover (Xie et al., 2020). Notably, GABA and Pro contents increased more under DS condition than WW condition (**Figure 4**), suggesting that AMF might enhance drought resistance by promoting GABA and Pro accumulation under DS condition (Mekonnen et al., 2016; Qian et al., 2018). Furthermore, AMF colonization increased Glu, Gln, Asp, Lys, and Arg contents (**Figure 4**), potentially enhancing N assimilation and storage within plants, thus promoting the synthesis of other AAs (Yang et al., 2020; Hildebrandt et al., 2015; Winter et al., 2015). The increase in Thea content, directly associated with tea quality (Lin et al., 2023), in AMF-colonized seedlings further indicates the beneficial effect of AMF inoculation on tea quality.

### 4.4 Changes in N metabolism-related enzymes activities induced by AMF

NR activity in tea plants is a key determinant of NO_3_- reduction ability (Liu et al., 2022b). DS treatment significantly suppressed NR activity, consistent with previous findings (Du et al., 2020; Zahoor et al., 2017). However, after AMF inoculation, NR activity was significantly increased in both leaves and roots (**Figure 5**), while PCA results showed a positive correlation between NR activity and N content in both leaves and roots (**Figures 8**, **9**), suggesting that AMF can enhance NO_3_^-^ reduction by increasing NR activity, thereby promoting N absorption by roots. Additionally, DS treatment inhibited GOGAT and GDH activities, but these were enhanced after AMF inoculation (**Figure 5**). As a key enzyme in the GS-GOGAT cycle, GS activity was significantly reduced in non-AMF seedlings under DS condition but increased in AMF-colonized seedlings regardless of water status (**Figure 5**). AMF inoculation had no significant impact on GS activity in roots (**Figure 5B**), possibly due to the weak influence of a single AMF (Xie et al., 2021). Nevertheless, AMF plays a significant role in enhancing N assimilation in tea seedlings.

DS treatment significantly inhibited GOT and GPT activities in leaves and roots, whereas AMF inoculation significantly alleviated this inhibition (**Figure 5**), similar to findings on exogenous spermidine effects on maize GOT and GPT under DS condition (Dong et al., 2022). This suggests that AMF may alleviate N metabolic disorders caused by DS by enhancing transaminase activity (Dong et al., 2022). DS treatment also significantly reduced root GAD activity and leaf GAD activity in AMF-colonized seedlings (**Figure 5**). However, AMF inoculation significantly enhanced GAD activity in both leaves and roots, consistent with GABA content changes (**Figure 5**). Meanwhile, PCA results indicated that root GAD activity was positively correlated with GABA content (**Figure 9**), suggesting that AMF may promote GABA accumulation by enhancing GAD activity, thereby enhancing drought resistance (Mekonnen et al., 2016). Furthermore, AMF inoculation alleviated the inhibitory effect of DS treatment on ASNS activity (**Figure 5**), consistent with Asn content changes (**Figure 4**), while the results of PCA also indicated a positive correlation between ASNS activity and Asn content in both leaves and roots (**Figure 8**; **Figure 9**), indicating that AMF may enhance Asn content by enhancing ASNS activity, mitigating DS’s inhibitory effect on N transportation (Gaufichon et al., 2010; Li et al., 2016b). The increase in PK activity and suppression of PDH and PDC activities induced by DS treatment might increase pyruvate content (**Figure 5**), potentially inducing increased respiratory activity as a physiological response to DS treatment (Zabalza et al., 2009). Additionally, the activities of PEPC, PK, PDC, PDH, and PDK (except PDK under DS treatment) in both leaves and roots were significantly enhanced by AMF treatment (**Figure 5**), potentially influencing other metabolic reactions (Jardine et al., 2010).

### 4.5 N metabolism-related enzyme genes expression pattern in mycorrhizal colonized seedlings

Regarding the expressions of N assimilation-related genes in tea roots, DS treatment significantly downregulated the expressions of *CsNR*, *CsNiR*, *CsGs1*, *CsGs2*, *CsFd-GOGAT*, *CsGDH1*, and *CsGDH2* in non-AMF seedlings (**Figure 7B**), consistent with previous findings (Xia et al., 2020). However, DS treatment had varying effects on these genes in AMF-colonized seedlings (**Figure 7B**), suggesting AMF involvement in multiple pathways. Under WW condition, AMF inoculation significantly upregulated the expressions of *CsNR*, *CsGS1*, and *CsNADH-GOGAT*, while under DS condition, AMF colonization upregulated the expressions of *CsNiR*, *CsGS1*, *CsGS2*, *CsNADH-GOGAT*, *CsGDH1*, and *CsGDH2*, consistent with changes in N metabolism-related enzymes activities (**Figure 7B**). Meanwhile, PCA results also showed that the expression of *CsNR* was positively correlated with NR activity, and the expressions of *CsGS1* and *CsGS2* were positively correlated with GS activity (**Figure 9**). These genes and enzymes are closely related to N assimilation, indicating that AMF may alleviate DS’s inhibitory effect on N assimilation by regulating the expressions of N assimilation-related genes and promoting the activities of N metabolism enzymes. Notably, AMF inoculation downregulated *CsGDH1* and *CsGDH2* expressions under WW condition (**Figure 7B**), possibly due to the synergistic role of *CsGSs* and *CsGDHs* in ammonium assimilation (Tang et al., 2021). This study also showed that AMF inoculation upregulated the expressions of *CsGSs* and *CsNADH-GOGAT* (**Figure 7B**), potentially promoting NH_4_^+^ assimilation while weakening *CsGDHs*’ function (Tang et al., 2021). In tea leaves, DS induced overexpression of *CsNiR* (**Figure 7A**), potentially a stress response to alleviate NO_2_^-^ accumulation toxicity under DS condition (Ezzine and Ghorbel, 2006). Alternatively, the downregulation of *CsGSs* under DS treatment could lead to NH_4_^+^ accumulation and upregulation of *CsGDHs*, promoting NH_4_^+^ assimilation and positive regulation of *CsNiR* expression to meet N requirements (Xia et al., 2020; Tang et al., 2021). The regulation of AMF inoculation on the expression of leaf N metabolism-related genes is relatively complex (**Figure 7A**). Under WW condition, AMF inoculation significantly upregulated *CsNiR*, *CsFd-GOGAT*, and *CsGDH1* expressions while downregulating *CsNR*, *CsGS1*, *CsNADH-GOGAT*, and *CsGDH2* expressions (**Figure 7A**). Under DS condition, AMF colonization upregulated *CsNR*, *CsGS1*, *CsGDH1*, and *CsGDH2* expressions, while downregulating *CsNiR*, *CsGS2*, *CsNADH-GOGAT*, and *CsFd-GOGAT* expressions (**Figure 7A**). These changes suggest that AMF influences N metabolism comprehensively, potentially regulating multiple genes to modulate enzymes activities (Chen et al., 2023).

The expressions of Thea metabolism-related genes are crucial for its accumulation (Liu et al., 2020b). This study revealed that DS treatment significantly downregulated the expressions of *CsTS1*, *CsTS2*, *CsGGT1*, *CsGGT3*, and *CsSAMDC* in the roots of tea seedlings (**Figure 7B**), which corresponded with reduced Thea content, consistent with Wang et al. (2016). This indicates that DS treatment negatively affects Thea accumulation. Notably, AMF inoculation significantly upregulated the expressions of *CsTS2*, *CsGGT1*, and *CsADC* regardless of water conditions (**Figure 7B**), aligning with the increase in Thea content in roots (**Figure 4B**). Meanwhile, the results of PCA showed strong correlation between CsTS2, CsGGT1 and Thea content (**Figure 9**), indicating that these genes are crucial for Thea synthesis and that AMF symbiosis can enhance Thea synthesis in roots.

In leaves, DS significantly downregulated the expressions of *CsTS1*, *CsTS2*, *CsSAMDC*, *CsADC*, and *CsCuAO* while upregulating the expressions of *CsALT* and *CsGGT1* (**Figure 7A**). The significant downregulation of *CsSAMDC* and *CsADC*, which function similarly to *CsAlaDC*, could hinder EA synthesis, a precursor for Thea (Lin et al., 2023; Shi et al., 2011). Consequently, Thea content in leaves decreased despite the upregulation of *CsGGT1* expression (**Figure 4A**), suggesting that the synergistic action of *CsAlaDC* and *CsTS* is crucial for high-concentration Thea synthesis in tea plants (Zhu et al., 2021). AMF colonization reasonably upregulated the expressions of *CsGGT1*, *CsGGT3*, and *CsADC* to varying degrees (**Figure 7A**), while PCA results also showed that CsGGT3 and CsADC were positively correlated with Thea content (**Figure 8**), confirming AMF’s stimulatory role in Thea synthesis. However, AMF inoculation significantly downregulated *CsPAO* expression, despite its crucial role in plant growth, development, and stress resistance (Yu et al., 2019), warranting further research.

## 5 Conclusion

In conclusion, our comprehensive study of the interplay between DS and AMF inoculation in tea seedlings has yielded insightful findings. DS was found to significantly hinder mycorrhizal infection, increasing mycorrhizal dependency and negatively impacting key physiological processes, including biomass accumulation, AAs content, and N metabolism enzyme activities in both roots and leaves. This ultimately led to a reduction in tea quality attributes such as tea polyphenol content and Thea content. Conversely, AMF inoculation emerged as an effective countermeasure, significantly boosting leaf and root biomass, N content, and AAs content, while enhancing the accumulation of tea polyphenols and catechins. Furthermore, AMF promoted the expression of genes and activities of enzymes involved in N metabolism, facilitating efficient nitrogen assimilation and alleviating the adverse effects of DS on the growth and development of tea plants. Our results underscore the potential of AMF as a biological tool to enhance tea plant resilience against DS, with implications for sustainable tea production under changing climatic conditions. Further research is warranted to elucidate the underlying molecular mechanisms and to optimize AMF inoculation strategies for wide-scale application in tea cultivation.

## Data Availability

The original contributions presented in the study are included in the article/supplementary material. Further inquiries can be directed to the corresponding authors.
